# Low cardiac dose and neutrophil-to-lymphocyte ratio predict overall survival in inoperable esophageal squamous cell cancer patients after chemoradiotherapy

**DOI:** 10.1038/s41598-021-86019-2

**Published:** 2021-03-23

**Authors:** Yu-Chieh Ho, Yuan-Chun Lai, Hsuan-Yu Lin, Ming-Hui Ko, Sheng-Hung Wang, Shan-Jun Yang, Po-Ju Lin, Tsai-Wei Chou, Li-Chung Hung, Chia-Chun Huang, Tung-Hao Chang, Jhen-Bin Lin, Jin-Ching Lin

**Affiliations:** 1 Department of Radiation Oncology, Changhua Christian Hospital, Changhua, 500 Taiwan; 2 Division of Medical Physics, Department of Radiation Oncology, Changhua Christian Hospital, Changhua, 500 Taiwan; 3 Division of Hematology/Oncology, Department of Internal Medicine, Changhua Christian Hospital, Changhua, 500 Taiwan; 4 Department of Radiation Oncology, Lukang Christian Hospital, Changhua Christian Medical Foundation, Lukang, 505 Taiwan; 5 Department of Medical Imaging Radiological Science, Central Taiwan University of Science and Technology, Taichung, 406 Taiwan; 6 Department of Medical Imaging and Radiological Technology, Yuanpei University of Science and Technology, Hsinchu, 300 Taiwan; 7 Division of Translation Research, Research Department, Changhua Christian Hospital, Changhua, 500 Taiwan; 8 Institute of Clinical Medicine, School of Medicine, National Yang-Ming University, Taipei, 112 Taiwan

**Keywords:** Cancer, Gastrointestinal cancer, Oesophageal cancer

## Abstract

We aimed to determine the prognostic significance of cardiac dose and hematological immunity parameters in esophageal cancer patients after concurrent chemoradiotherapy (CCRT). During 2010–2015, we identified 101 newly diagnosed esophageal squamous cell cancer patients who had completed definitive CCRT. Patients' clinical, dosimetric, and hematological data, including absolute neutrophil count, absolute lymphocyte count, and neutrophil-to-lymphocyte ratio (NLR), at baseline, during, and post-CCRT were analyzed. Cox proportional hazards were calculated to identify potential risk factors for overall survival (OS). Median OS was 13 months (95% confidence interval [CI]: 10.38–15.63). Univariate analysis revealed that male sex, poor performance status, advanced nodal stage, higher percentage of heart receiving 10 Gy (heart V10), and higher NLR (baseline and follow-up) were significantly associated with worse OS. In multivariate analysis, performance status (ECOG 0 & 1 vs. 2; hazard ratio [HR] 3.12, 95% CI 1.30–7.48), heart V10 (> 84% vs. ≤ 84%; HR 2.24, 95% CI 1.26–3.95), baseline NLR (> 3.56 vs. ≤ 3.56; HR 2.36, 95% CI 1.39–4.00), and follow-up NLR (> 7.4 vs. ≤ 7.4; HR 1.95, 95% CI 1.12–3.41) correlated with worse OS. Volume of low cardiac dose and NLR (baseline and follow-up) were associated with worse patient survival.

## Introduction

Esophageal cancer ranks seventh in global cancer incidence (572,000 new cases) and sixth in overall mortality (509,000 deaths), and eastern Asia has the highest reported incidence of esophageal cancer^1^. In Taiwan, squamous cell carcinoma is the predominant type of esophageal cancer, and it continues to increase, probably due to an increase in smoking, alcohol consumption, and betel quid chewing^2,3^. Esophageal cancer is an aggressive disease and often harbors dismal survival outcomes^4–7^. Definitive concurrent chemoradiotherapy (CCRT) is the standard treatment for unresectable locally advanced esophageal cancer^7–9^. Apart from the neoadjuvant CCRT followed by surgery, the purposed radiation dose to the gross disease in the definitive setting will be at least 50.4 Gy^9^, and even higher with modern radiation techniques^10^.

Traditionally, we focused on the radiation dose to the target volume, in terms of the gross tumor burden, to evaluate further disease control and survival outcomes^9^. With modern methods to deliver radiation, a simultaneous integrated radiotherapy dose boost to the gross tumor and nodal disease is well tolerated and may improve local control and even overall survival (OS)^10^. Apart from the simultaneous dose escalation, we can also evaluate dosimetric parameters of organs at risk (OAR), especially the heart and lung, to elucidate the possible effect of survival outcomes. Past studies have reported that an increase in radiation dose to OAR is associated with worse survival in the lung and esophageal cancer^11–17^.

There are limited biomarkers to predict the disease status or prognosis in esophageal cancer; however, the need for blood parameters indicative of systemic inflammation and immunity is of great concern because of the possible survival correlations^18–26^. Absolute neutrophil count (ANC), absolute lymphocyte count (ALC), or the ratio between, i.e., the neutrophil-to-lymphocyte ratio (NLR), can serve as simple indicators of systematic response to cancer cells and further improve treatment response. Testing in the pre-treatment, during, or post-treatment settings will represent the dynamic responses of those blood parameters. Despite chemotherapy, the radiation-induced lymphopenia during CCRT will also contribute to the changes in those parameters, including survival outcomes^15,27–33^. This study aimed to evaluate the possible prognostic factors and normal tissue dosimetric parameters affecting the OS in non-operable patients who had completed the whole course of radiotherapy.

## Results

### Patient outcomes and causes of death

Patient, tumor, and treatment characteristics are summarized in Tables 1 and 2. The majority of the patients were current or former smokers (84%), alcohol drinkers (90%), and betel quid users (67%). Most patients underwent a positron emission tomography/computed tomography (PET/CT; 74%) scan as part of their initial staging workup. All patients had squamous cell carcinoma, with the majority having moderately differentiated carcinoma (70%) and therefore received photon radiation with either a three-dimensional conformal radiation therapy (3DCRT) (21.8%), intensity-modulated radiation therapy (IMRT) (42.6%), or volumetric-modulated arc therapy (VMAT) (35.6%). More than one-quarter (27.7%) of patients received image-guided radiation therapy (IGRT), with online cone-beam CT-based imaging correction before treatment. The median prescribed dose was 60 Gy (range 48.6–74 Gy).

**Table 1:** Patient and tumor characteristics.

Patient and tumor characteristics (n = 101)	Median	Range or %
**Sex**		
Men	91	90.1
Women	10	9.9
Age (years)	60	38–88
Pre-treatment body weight (kg)	57	36–91
**Smoker**		
No (never smoked)	14	13.9
Yes (current smoker or quitted)	75	74.3
Unknown	12	11.9
**Alcohol drinker**		
No (never or not regular)	9	8.9
Yes (current use or ever regularly use)	79	78.2
Unknown	13	12.9
**Betel-nuts chewer**		
No (never or not regular)	28	27.7
Yes (current use or ever regularly use)	56	55.4
Unknown	17	16.8
**ECOG PS, n (%)**		
0 and 1	92	91.1
2	9	8.9
ACCI, excluding esophageal cancer	2	0–6
Esophageal tumor length, centimeter	6	2–25
**Location, n (%)**		
Cervical esophagus	8	7.9
Upper esophagus	21	20.8
Middle esophagus	38	37.6
Lower esophagus	34	33.7
**Histologic grade, n (%)**		
Grade 1	2	2.0
Grade 2	71	70.3
Grade 3	8	7.9
Unknown	20	19.8
**Clinical T stage, n (%)**		
T1 and T2	36	35.6
T3 and T4	65	64.4
**Clinical N stage, n (%)**		
N0 and N1	44	43.6
N2 and N3	57	56.4
**cTNM stage, n (%)**		
II	26	25.7
III	75	74.3

*ECOG PS* Eastern Cooperative Oncology Group Performance Status, *ACCI* age adjusted Charlson’s comorbidity index.

**Table 2:** Treatment modalities.

Treatment modalities (n = 101)	Median	Range or %
**RT technique**		
3D-CRT	22	21.8
IMRT	36	35.6
IG-IMRT	7	6.9
VMAT	15	14.9
IG-VMAT	21	20.8
Radiation dose (Gy)	60	48.6–74
Number of fractions	30	25–37
**Dose-volume of heart (%)**		
V10	92	0–100
V20	45	0–98
V30	16	0–53
V40	5	0–23
**Dose-volume of lung (%)**		
V5	92	29–100
V10	70	21–96
V20	22	6–48
Mean heart dose (cGy)	2059	5–3174
Mean lung dose (cGy)	1516	580–2232
**Radiotherapy and chemotherapy sequence, n (%)**		
Induction chemo, CCRT, and ± adjuvant chemo	53	52.5
CCRT and ± adjuvant chemo	46	45.5
Sequential RT and chemo	2	2.0
**CCRT chemotherapy regimen**		
Cisplatin and fluorouracil (PF)	95	94.1
Carboplatin and fluorouracil	2	2.0
Weekly cisplatin	2	2.0
Sequential PF and RT	2	2.0

*Vx(%)* relative percent of volumes for at least x (Gy), *3DCRT* 3-dimensional conformal radiation therapy, *IMRT* intensity-modulated radiation therapy (IMRT), *VMAT* volumetric modulated arc therapy, *IG* image-guided, *CCRT* concurrent chemoradiation.

Almost all patients (98%) received concurrent chemotherapy with RT (CCRT), and only two patients had chemotherapy and RT sequentially. Fifty-three (52.5%) patients also received induction chemotherapy. The mainstay chemotherapy regimen was triweekly cisplatin and fluorouracil (96%). Patients received induction chemotherapy in a median number of one cycle (range 0–2) and received CCRT with a median number of one cycle of cisplatin–fluorouracil (range 0–3), based on the patients' tolerance and the chosen chemotherapy regimen. During the CCRT, the most acute toxicity of grade ≥ 3 was hematological toxicity, and the detailed analysis was shown separately. The other grade 3 toxicity included dysphagia (6%), mucositis (2%), anorexia (1%), and fatigue (1%), and there was no reported grade ≥ 4 toxicity.

The Median follow-up duration was 13 months (range 3–104 months) in all patients and 60 months (range 52–104 months) in survivors. The estimated median OS was 13 months in all patients (95% CI 10.38–15.63), DSS (disease-specific survival) was 14 months (95% CI 11.60–16.40), and PFS (progression-free survival) was 9 months (95% CI 7.70–10.30). The estimated 2-year FFDM (freedom from distant metastasis) and FFLR (freedom from locoregional recurrence) were 37.1% and 40.3%, respectively.

By the last follow-up, 88 patients (87%) had died; 79 patients (90%) died due to disease progression or subsequent complications. Others died due to second primary cancer (5%), chronic obstructive pulmonary disease (2%), tuberculosis infection (1%), and unknown etiology (2%). Cardiac complications along with acute myocardial infarction and life-threatening arrhythmia were noted in four of the expired patients (5%), and no survivor reported newly diagnosed cardiac disease. Further analysis divided heart V10 into two groups with survival significance, we presented the cause of mortality in each group and found no notable difference in between (see Supplementary Table S2 online).

### Hematological parameters and toxicity

Data of pre-treatment baseline complete blood count (CBC) were available in 95 patients. Median days between baseline CBC and RT were 18 days (range: 2–81). Median baseline ANC, ALC, and NLR data were 5384 cells/mm^3^ (range: 1716–14,309), 1635 cells/mm^3^ (range: 512–4127), and 3.56 (range: 0.77–13.92), respectively. Notably, there were 13/95 (14%) patients with a low baseline ALC level: eight patients with grade 1 lymphopenia and five patients with grade 2 lymphopenia.

Data of the ALC nadir and the highest NLR were available for all patients. The ALC nadir and the highest NLR occurred at the same median of 28 days (range: 7–74 and 1–74, respectively) after RT start, respectively. There were 28 patients with different sampling days in between the ALC nadir and the highest NLR. The median nadir ALC and the highest NLR were 223.2 cells/mm^3^ (range: 16–742.1) and 17.4 (range: 2.64–155), respectively. There were 10/101 (10%) patients with grade 2 lymphopenia, 47 (47%) with grade 3, and 44 (44%) with grade 4 lymphopenia at the nadir. The median decreased ALC percentage (%), from baseline to the nadir, was 86.18 (range: 39.17–98.76).

At a median of 41 days (range: 29–109) after RT finished, 86 patients were available for a follow-up CBC. The median ANC, ALC, and NLR data were 3539 cells/mm^3^ (range: 468–27,424), 820 cells/mm^3^ (range: 36–3431), and 4.51 (range: 0.59–48.75), respectively. Seventeen (20%) patients remained at grade 1 lymphopenia, 25 (29%) patients with grade 2, 15 (17%) patients with grade 3, and 2 patients (2%) with grade 4 at the follow-up.

### Univariate and multivariate models of OS

Factors associated with OS on univariate analysis are summarized in Table S1. The entire list of factors can be found as Supplementary Table S1 online. Male sex, performance status, pre-treatment body weight, smoking or alcohol drinking history, primary esophageal tumor size, advanced nodal staging and clinical staging, heart dosimetric parameters, pre-treatment lymphopenia or not, baseline NLR, and highest NLR were significantly associated with worse OS. Notably, the baseline, nadir, and recovery ALC levels were not associated with OS. In the selected multivariate Cox regression model shown in Table 3, poor performance status with ECOG = 2 (HR 3.116, 95% CI 1.298–7.481), heart V10 > 84% (HR 2.235, 95% CI 1.264–3.950), baseline NLR > 3.56 (HR 2.357, CI 1.387–4.004), and follow-up NLR > 7.4 (HR 1.951, CI 1.115–3.414) remained significantly associated with worse OS.

**Table 3:** Univariate and multivariate Cox regression of clinical, hematologic, and dosimetric variables associated with overall survival.

Variables	Univariate			Multivariate		
	P value	HR	95% CI	P value	HR	95% CI
**Sex**						
Man	Ref			Ref		
Woman	**0.028**	0.361	0.145–0.894	0.134	0.477	0.181–1.256
**ECOG performance status**						
0 and 1	Ref			Ref		
2	**0.035**	2.117	1.053–4.256	**0.011**	3.116	1.298–7.481
**N stage**						
N0 and N1	Ref			Ref		
N2 and N3	**0.065**	1.502	0.975–2.315	0.723	1.095	0.664–1.804
**Heart V10 (%)**						
≤ 84	Ref			Ref		
> 84	**0.019**	1.835	1.107–3.040	**0.006**	2.235	1.264–3.950
**Baseline NLR**						
≤ 3.56	Ref			Ref		
> 3.56	**0.007**	1.835	1.181–2.851	**0.002**	2.357	1.387–4.004
**Follow-up NLR**						
≤ 7.4	Ref			Ref		
> 7.4	**0.030**	1.774	1.059–2.974	**0.019**	1.951	1.115–3.414

### Higher heart V10, baseline NLR, and follow-up NLR associated with worse OS

Kaplan–Meier analysis was used for further survival evaluation for the heart V10, baseline NLR, and follow-up NLR.

**Figure 1:**
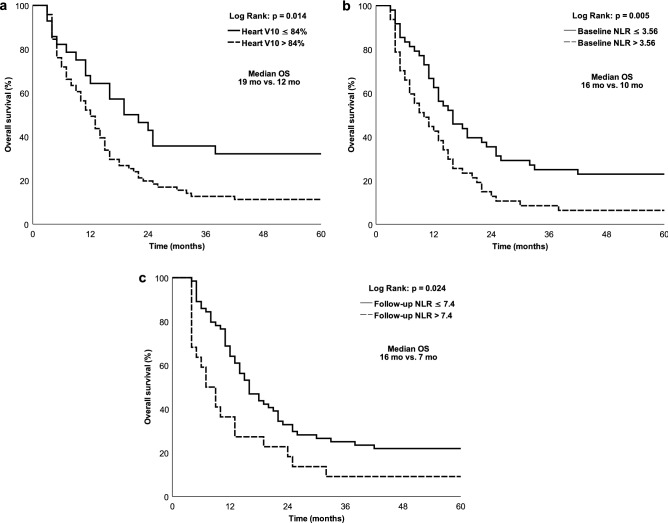
Kaplan–Meier’s curves for OS (overall survival), and patients are stratified by (**a**) heart V10 as > 84% (dotted line) or ≤ 84% (solid line); (**b**) baseline NLR as > 3.56 (dotted line) or ≤ 3.56 (solid line); (**c**) follow-up NLR as > 7.4 (dotted line) or ≤ 7.4 (solid line).

Median OS rates stratified by heart V10 > 84% and ≤ 84% were 12 months and 19 months, respectively (p = 0.014, Fig. 1a). The estimated 2-year OS rates stratified by heart V10 > 84% and ≤ 84% were 19.7% and 42.9%, respectively. Heart V10 > 84% was also associated with worse PFS (p = 0.005), DSS (p = 0.01) and FFDM (p = 0.021), but was not associated with FFLR (see Supplementary Fig. S1–S3a online).

The median OS rates stratified by baseline NLR > 3.56 and ≤ 3.56 were 10 months and 16 months, respectively (p = 0.005, Fig. 1b). The estimated 2-year OS rates stratified by NLR > 3.56 and NLR ≤ 3.56 were 12.8% and 35.4%, respectively. Baseline NLR > 3.56 was also associated with worse PFS (p = 0.007), DSS (p = 0.006), and FFDM (p = 0.002) but not associated with FFLR (see Supplementary Fig. S1–S3b online).

Further median OS rates stratified by follow-up NLR > 7.4 and ≤ 7.4 were 7 months and 16 months, respectively (p = 0.024, Fig. 1c). The estimated 2-year OS stratified by NLR > 7.4 and ≤ 7.4 were 18.2% and 32.8%, respectively. Follow-up NLR > 7.4 was also associated with worse PFS (p = 0.024) and DSS (p = 0.011) but not associated with FFDM or FFLR (see Supplementary Fig. S1–S2c online).

## Discussion

This study demonstrated that radiation heart dose–volume and baseline and follow-up NLR correlated with survival outcomes in esophageal cancer patients receiving non-operative definitive treatments with radiotherapy and chemotherapy. Each parameter had its cut-off values to prevent mortality, i.e., heart V10 ≤ 84%, baseline NLR ≤ 3.56, and follow-up NLR ≤ 7.4, respectively. Moreover, they correlated with the other different survival outcomes, despite the FFLR. However, the lung dosimetric parameter in our analysis was not associated with survival outcomes.

Limiting the heart dose in the thoracic radiotherapy is never a new concept. Since the long-term follow-up data from breast cancer and lymphoma^34–37^, we have learned about the relationship between heart dose and further cardiac complication. However, Darby et al. reported that more than half of the patients developed major coronary events for more than 10 years after breast cancer was diagnosed^37^. In unresectable locally advanced esophageal cancer, the survival outcomes are dismal^10^, compared to those in breast cancer and lymphoma. In lung cancer, which harbored a similar thoracic radiation field, the heart dose may independently be associated with poor OS^13–15,38^. There are limited data about the relationship between heart dose and survival outcomes in esophageal cancer, and heart V30 > 45% is found to be independently associated with worse survival^16^. Moreover, based on the data for lung cancer, the RTOG 0617 trial suggested heart V5 and heart V30 were associated with an increased risk of death^39^. The higher heart V50 independently predicted worse survival and 25% was used as stratification for 2-year OS rates with 45.9% versus 26.7% (p < 0.0001)^14^. We presented our data from patients with pure squamous cell carcinoma histology and disclosed heart V10 as the strongest heart dosimetric variable to correlate with OS. When stratified with 84%, the predicted OS had approximately 7 months in differential; the 2-year OS rates were 19.7% versus 42.9% (p = 0.014). The other endpoints, including PFS, DSS, and FFDM, were also with significant differences. Although three out of four cardiac complications were in the group of Heart V10 > 84%, the exact incidence of symptomatic cardiac disease is still reasonably low in patients with such limited survival lifespans^40–42^. Unintentional cardiac radiation may cause more than simple cardiac complications.

Neutrophil, the most abundant immune cell population and traditionally regarded as indispensable antagonists of microbial infection and facilitators of wound healing, may play an essential role in the cancer setting^18^. Lymphocyte, especially cytotoxic T lymphocyte, is critical in mediating cellular immunity against neoplastic cells and causing further progression and metastasis^43–45^. Clinically, the systemic inflammatory immune dynamics, presented as NLR, has been introduced to predict poor survival outcomes in esophageal cancer^23,25^. It represented the ratio of circulating neutrophils to lymphocytes and was supposed to correlate with the interaction between inflammation and immunity in the patient. Although NLR could not have the same behavior as tumor microenvironment, it showed some clinical significance as the general inflammation-immunity condition at the time of sampling, and warranted further preclinical and translational investigations. In the setting of CCRT, a cut-off value of 2.64 for pre-treatment NLR predicts OS in esophageal squamous cell carcinoma^22^, and a cut-off of 2.43 may be the optimal value for survival after surgical treatment^46^. The optimal cut-off point of NLR varies between studies, and after reviewing a total of 20 studies, it was noted to range from 1.7 to 5^23^. We found 3.56 as the optimal predicting value to median OS (p = 0.005). It also predicts worse PFS, DSS, and FFDM. Although the highest NLR during CCRT showed no correlation with OS, we found that follow-up NLR > 7.4 after CCRT remained independently correlated with poor OS, PFS, and DSS. There are scarce studies on the relationship between survival and follow-up NLR, and it may represent prolonged inflammation with decreased immunity. In the present study, we had set the analytic timing of follow-up NLR with at least one month and tried to diminish the influence of radiation. Prolonged elevated NLR may reflect constitutional immunity response, due to an infection, or residual tumor-related immunosuppression. In patients who received surgical treatment, time-dependent dynamic changes in NLR during neoadjuvant CCRT or postoperative may predict treatment response and survival ^24,26^.

With thoracic radiation, the cardiac dose correlated with immunosuppression and poor survival in lung cancer, and the reported heart V50 and higher NLR at 4 months post-RT started with reduced OS. Heart V50 > 25% was also associated with NLR > 10.5 at 4 months post-RT^15^. There is a delicate model to estimate the exposure of circulating immune cells by mean lung, heart, liver, integral body region, dose, and the number of fractions. The higher effective dose to circulating immune cells (EDIC) correlated with elevated thoracic radiation dose with more than grade 4 lymphopenia and worse OS, PFS, and distant metastasis-free survival (DMFS)^32^. Lymphocytes are the most radiosensitive cell type in the body^47^ and the only non-dividing cells killed by small doses of X-rays^48^. Even a 2 Gy irradiation could reduce the population by 50% over in vitro assays^49^. Thus, the volume of the low cardiac dose can be immunosuppressive and even detrimental to survival outcomes. We found that the more the volume of heart receiving a low dose of RT, the more the survival outcomes worsened. We hypothesize that the radiation-induced immunosuppression indirectly contributes to mortality without overt symptomatic cardiac disease. It plays a role even at a low dose and may weigh more than those structural damages, especially in diseases with a limited survival time. To the best of our knowledge, this is the first study to emphasize the volume of low cardiac dose of RT with survival benefits. It may be a simple way to substitute the proposed model. Although there were factors correlating with heart V10, it still stands out after a multivariate Cox regression model. We believe it is worth our attention.

This study had some limitations due to the single-institute retrospective design. First, it only included a small number of patients and squamous cell carcinoma; other histologic subtypes may not be suitable. Second, we presented our institute's dosimetric data; although we have arrived at consensus about contouring in between, it may still be variable to each physician. We recorded only the gross structure of each organ without further substructure information^50^. Although the results to evaluate the whole heart as a blood pool may not be influenced, if there are detailed structure data, then subsequent analysis will be possible. Third, the hematological data were retrospectively obtained from clinical samples tested for CBC, and we did not test every patient with the same interval during or post-CCRT. Moreover, other factors that may potentially influence the blood counts during or post-CCRT, such as infections or non-chemotherapy medications, may need to be taken into consideration. However, as lymphocytes remain the most radiosensitive cells, we included only patients who had completed the whole scheduled RT course and limited the possible confounder.

Despite many limitations, we did demonstrate that a higher cardiac dose, especially the heart V10, and higher baseline or follow-up NLR, was related to poor survival in squamous cell esophageal cancer. Our findings need verification through further prospective studies with more focus on dosimetric data, not only on the gross tumor but those surrounding blood pools. We suggest a greater interest in the cardiac dose, instead of the lung dose, in the setting of non-operative treatments. Despite clinical judgment to optimize the prescription or treatment planning, advancement in radiotherapy technique from photon to proton, is an alternative way to further reduce the volume of irradiated normal organ. Moreover, elevated NLR at baseline or follow-up periods warrant treatment intensification by novel approaches.

## Methods

### Patient characteristics and study design

This study is a single-institution retrospective review of 101 patients with non-metastatic esophageal cancer, treated with non-surgical treatments including definitive radiotherapy (RT) with or without induction, concurrent, and adjuvant chemotherapy. Patients were diagnosed between 2010 and 2015 and included if they received a completed course of radiotherapy, at a median total radiation dose of 60 Gy (range: 48.6–74 Gy) with standard daily fractionation (1.8–2.0 Gy per fraction), and had complete blood counts (CBC) check-ups before, during, or after RT.

All patients had esophagogastroduodenoscopy (EGD) biopsy-proven squamous cell carcinoma, calculated the gross tumor size under the scope, and staged by the 7^th^ edition of the Union for International Cancer Control/American Joint Committee on Cancer TNM classification system with a chest CT scan. Bronchoscopy was used to evaluate the trachea whether there was any suspicious direct invasion from the chest CT scan. Metastatic disease study with whole-body F-18 fluorodeoxyglucose PET/CT, Tc99m methylene diphosphonate bone scan, or abdominal sonography was an option in the initial and further follow-up workup based on the physician's decision. Those with any other cancer diagnosed or treated before this cancer, and those with synchronous cancer, were excluded. Age-adjusted Charlson comorbidity index (ACCI) score^51,52^ should be calculated (current esophageal cancer diagnosis not included) to estimate the 10-year pre-treatment risk of mortality.

Treatment-related toxicities were graded using the Common Terminology Criteria for Adverse Events (CTCAE), version 4.0. After treatment, follow-up included chest CT and EGD every 3–6 months to evaluate for local, regional, and distant failure, in combination with any other metastatic disease study if needed. The data source for this review was approved by the Institutional Review Board of the Changhua Christian hospital, and it waived the requirement for written informed consent of this study. (Approval number CCH IRB No.: 180310). It was confirmed that all procedures adhered to the relevant guidelines and regulations.

### Radiation treatment and dosimetric analysis

The dosimetric analysis was performed on all patients, with available RT plans on the pinnacle treatment planning system (Philips Radiation Oncology Systems, Fitchburg, WI). The target volume included primary tumor and any lymphadenopathy plus a 1-cm circumferential margin and a 3- to 5-cm longitudinal margin. Elective nodal irradiation may also be included in the target volume based on the physician's discretion. Planned target volume (PTV) and OAR, including heart and lung contours, were reviewed and recontoured (if needed) without adding a margin on each OAR. The dosimetric data were extracted by a dosimetrist and reviewed by a physician. The dose–volume histogram (DVH) parameter, as the heart and lung received the relative percent of volumes for at least **x** (Gy), was identified as **Vx** (%). The mean dose was also evaluated.

### Hematological parameters

Baseline ANC and ALC were analyzed before any treatment. ALC nadir was obtained with the lowest ALC level during RT, and the follow-up ANC and ALC were checked after a minimum of 28 days after RT completion. The baseline and follow-up neutrophil-to-lymphocyte ratio (NLR) was calculated by dividing the ANC by the ALC. The highest NLR during CCRT was obtained on the date with the lowest lymphocyte percentage on CBC.

### Statistical analysis

Continuous data are shown as the median and range, while categorical data are presented as numbers and percentages. Clinical endpoints included OS, PFS, DSS, FFDM, and FFLR. Follow-up time and time to clinical endpoints were calculated from the date of diagnosis.

Cox regression model was performed to find the possible associations of clinical, hematological, and dosimetric factors with OS, which was entered and tested in a forward-conditional manner. Variables with a p-value < 0.1 in univariate analysis were selected for multivariate analysis. If there are strong correlations between those variables, then we choose the factor with clinical significance. The first quartile of heart V10 and the median of baseline NLR were chosen thresholds to dichotomize continuous variables and to increase the specificity. Furthermore, the third quartile value of the follow-up NLR was also chosen for the same purpose. OS, PFS, DSS, FFDM, and FFLR rates were estimated with Kaplan–Meier analyses. The log-rank test was used to calculate the significance of survival estimate differences. We further tested the possible correlation of tumor characteristics and dosimetric parameters associated with the Heart V10 by Spearman's rank correlation coefficient (see Supplementary Table S3 online). A p-value ≤ 0.05 was considered statistically significant. Hazard ratios (HR) are reported with a 95% confidence interval (CI). All analyses were conducted and the figures related to survival curves were created using IBM SPSS version 25.0 (IBM Corp, Armonk, NY).

## Supplementary Information

Supplementary Information

## Data Availability

All data analyzed during this study are included in this published article (and its Supplementary Information files).
