# Supplementary material for: Low cardiac dose and neutrophil-to-lymphocyte ratio predict overall survival in inoperable esophageal squamous cell cancer patients after chemoradiotherapy
Source: Sci Rep. 2021 Mar 23;11:6644. doi: 10.1038/s41598-021-86019-2 (PMC7988072; doi:10.1038/s41598-021-86019-2)
Supplement: Supplementary file 1 — Supplementary Information [file 41598_2021_86019_MOESM1_ESM.pdf]

Volume of Low Cardiac Dose and Neutrophil-to-Lymphocyte Ratio Predict Overall Survival in Non-Operable Esophageal Squamous Cell Cancer After Chemoradiotherapy

Yu-Chieh Ho [1], Yuan-Chun Lai [2,5], Hsuan-Yu Lin [3], Ming-Hui Ko [2], Sheng-Hung Wang [4], Shan-Jun Yang [1], Po-Ju Lin [1], Tsai-Wei Chou [1], Li-Chung Hung [1], Chia-Chun Huang [1], Tung-Hao Chang [1,5,6], Jhen-Bin Lin [1,*] and Jin-Ching Lin [1,7,8]

Supplementary Figures

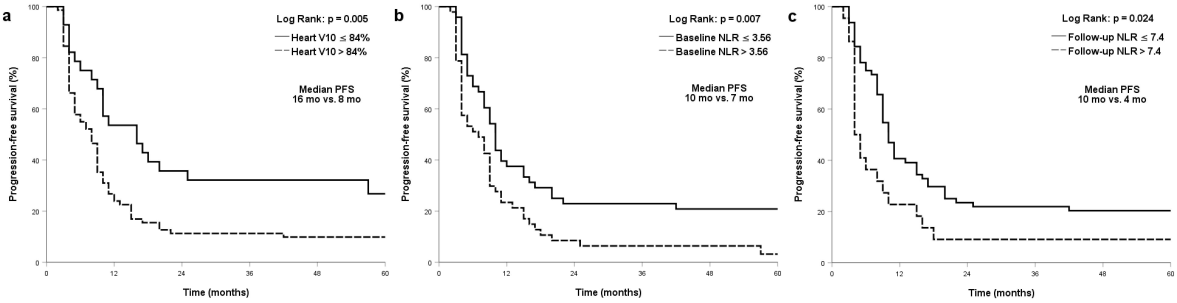

Figure S1. Kaplan–Meier's curves for PFS (progression-free survival), and patients are stratified by (A) heart V10 as >84% (dotted line) or ≤84% (solid line); (B) baseline NLR as >3.56 (dotted line) or ≤3.56 (solid line); (C) follow-up NLR as >7.4 (dotted line) or ≤7.4 (solid line).

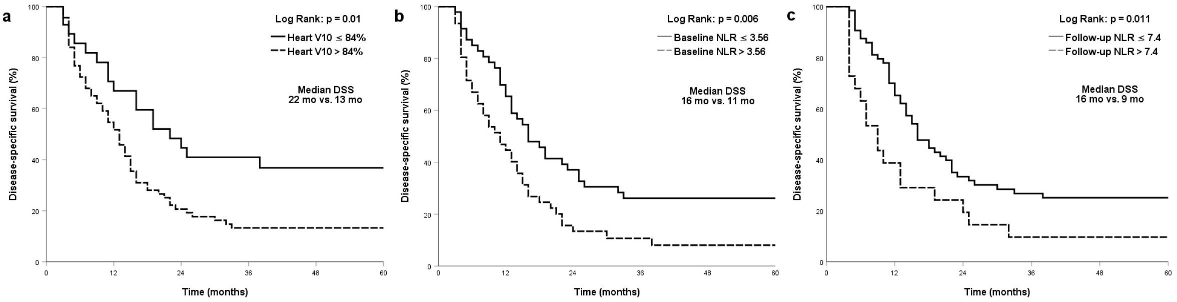

Figure S2. Kaplan–Meier's curves for DSS (disease-specific survival), and patients are stratified by (A) heart V10 as >84% (dotted line) or ≤84% (solid line); (B) baseline NLR as >3.56 (dotted line) or ≤3.56 (solid line); (C) follow-up NLR as >7.4 (dotted line) or ≤7.4 (solid line).

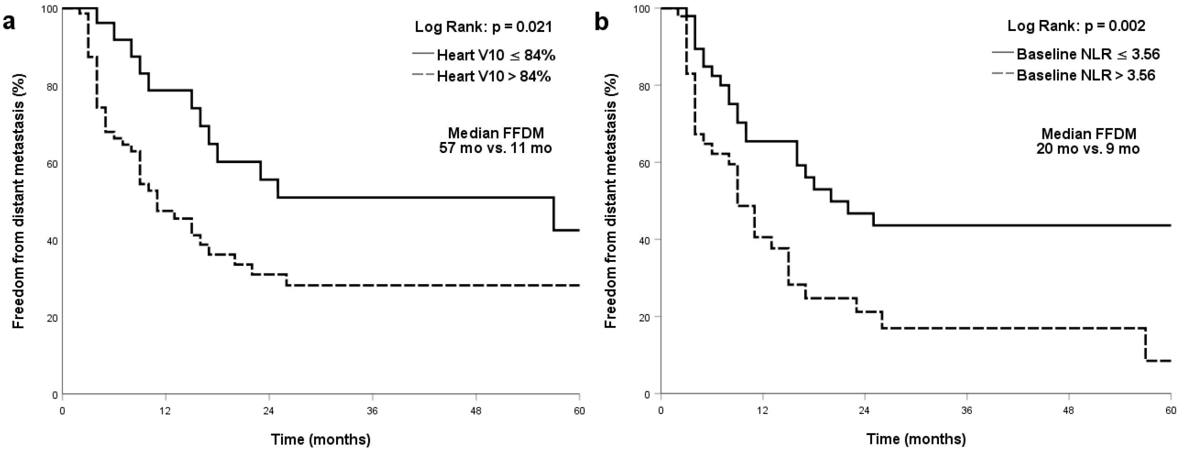

Figure S3. Kaplan–Meier's curves for FFDM (freedom from distant metastasis), and patients are stratified by (A) heart V10 as >84% (dotted line) or ≤84% (solid line); (B) baseline NLR as >3.56 (dotted line) or ≤3.56 (solid line).

Supplementary Tables

Table S1. Univariate Cox regression of clinical, hematologic, and dosimetric variables associated with overall survival

Patient characteristics	P value	HR	95% CI
Sex[b]			
Man	Ref		
Woman	**0.028**	0.361	0.145–0.894
Age	0.172	0.986	0.966–1.006
Age-adjusted CCI ≤3 vs. >3	0.818	0.935	0.526–1.660
Pre-Tx body weight (kg)[a]	**0.007**	0.975	0.958–0.993
ECOG performance status			
0 & 1	Ref		
2	**0.035**	2.117	1.053–4.256
Smoker or not[b]	**0.014**	2.532	1.210–5.298
Alcohol drinker or not[b]	**0.037**	2.934	1.066–8.074
Betel-nuts chewer or not	0.301	1.310	0.785–2.187
Tumor location<br>Cervical & U/3 vs. M & L/3	0.292	1.294	0.802–2.087
Tumor length (cm)[a]	**0.045**	1.062	1.001–1.126
Tumor Gr. 1 & 2 vs. 3	0.720	1.154	0.527–2.526
T Stage T1 & T2 vs. T3 & T4	0.115	1.430	0.917–2.231
N stage[c]			
N0 & N1	Ref		
N2 & N3	**0.065**	1.502	0.975–2.315
Clinical stage[c]			
Stage II	Ref		
Stage III	**0.099**	1.515	0.925–2.481
Prescribed RT dose (cGy)	0.855	0.997	0.963–1.032
3DCRT vs. IMRT or VMAT	0.658	0.897	0.553–1.455
IGRT or not	0.589	0.875	0.539–1.420
Heart Dmean[d]	**0.071**	1.000	1.000–1.001
Heart V10(%)[d]	0.113	1.008	0.998–1.017
Heart V10(%)[d]			
≤ 84	Ref		
> 84	**0.019**	1.835	1.107–3.040
Heart V20(%)[d]	**0.082**	1.008	0.999–1.018
Heart V30(%)[d]	**0.069**	1.015	0.999–1.031

Heart V40(%)[d]	**0.060**	1.035	0.999–1.074
Lung Dmean	0.282	1.000	1.000–1.001
Lung V5(%)	0.362	1.007	0.992–1.022
Lung V10(%)	0.346	1.006	0.994–1.019
Lung V20(%)	0.649	1.006	0.979–1.035
Cumulative Cisplatin dosage in CCRT	0.451	0.998	0.993–1.003
Cumulative Cisplatin dosage before CCRT finished	0.101	0.996	0.992–1.001
Baseline ALC	0.712	0.999	0.995–1.003
Baseline lymphopenia			
None	Ref		
Grade 1 or 2[a]	**0.043**	1.856	1.021–3.374
Baseline NLR[a]	**0.042**	1.091	1.003–1.188
Baseline NLR[a]			
≤ 3.56	Ref		
> 3.56	**0.007**	1.835	1.181–2.851
ALC nadir	0.206	0.993	0.982–1.004
Decreased ALC(%) at ALC nadir	0.218	1.012	0.993–1.030
Highest NLR[a]	**0.048**	1.007	1.000–1.014
Follow-up ALC	0.677	0.999	0.995–1.003
Follow-up NLR[d]	0.197	1.015	0.992–1.039
Follow-up NLR[d]			
≤ 7.4	Ref		
> 7.4	**0.030**	1.774	1.059–2.974

[a] *Due to the strong correlation of pre-treatment lymphopenia, baseline NLR, highest NLR, pre-treatment body weight, and primary esophageal tumor size, we chose the baseline NLR to further multivariate analysis.*

[b] *Male sex, smoking, and alcohol drinking history had a strong correlation in between; we chose sex for multivariate analysis.*

[c] *N stage and the clinical stage had a strong correlation; we chose the N stage, as with a numerically lower p-value, for multivariate analysis.*

[d] *There are strong correlations in heart dosimetric parameters, as Dmean, V10, V20, V30, & V40; we chose V10 for further analysis. Although the continuous variables of heart V10 fall short of significant association with OS, we found that if the heart V10 > 84%, as the first quartile of heart V10 in this cohort, had the most correlation with OS among all heart dosimetric parameters. The follow-up NLR had the same situation; we chose the third quartile value of the follow-up NLR (> 7.4) for further analysis.*

Table S2. Cause of mortality in patients stratified by heart V10 as >84% (n = 66) or ≤84% (n = 20)

Cause of mortality	**Herat V10 >84%**	**Heart V10 ≤84%**
Disease progression or subsequent complications *(such as infection or bleeding)*	59 (90%)	18 (90%)
--- with cardiac disease *(acute myocardial infarction or life-threatening arrhythmia)*	3 (5%)	1 (5%)
--- without cardiac disease	56 (85%)	17 (85%)
Second primary cancer	2	2
Chronic obstructive pulmonary disease	2	0
Tuberculosis infection	1	0
Unknown etiology	2	0

Table S3. Spearman's rank correlation coefficient of heart V10 >84% or ≤84% with tumor characteristics and dosimetric parameters

	Spearman's correlation coefficient (r)
Tumor characteristics	
**Tumor location**<br>Cervical & U/3 vs. M & L/3	**0.353, p < 0.001**
**Tumor length (cm)**<br>>6 (median length) vs. ≤6	**0.238, p = 0.049**
Tumor grade<br>Gr. 1 & 2 vs. 3	-0.055, p = 0.631
T Stage<br>T1 & T2 vs. T3 & T4	0.005, p = 0.963
N stage<br>N0 & N1 vs. N2 & N3	0.128, p = 0.205
Clinical stage<br>Stage II vs. Stage III	0.100, p = 0.327
**Prescribed RT dose (cGy)**	**-0.226, p = 0.025**
**3DCRT vs. IMRT or VMAT**	**-0.216, p = 0.032**
IGRT or not	-0.104, p = 0.307
Dosimetric parameters	
**Heart mean dose**	**0.751, p < 0.001**
**Heart V10**	**0.779, p < 0.001**
**Heart V20**	**0.683, p < 0.001**
**Heart V30**	**0.602, p < 0.001**
**Heart V40**	**0.491, p < 0.001**
**Lung mean dose**	**0.477, p < 0.001**
**Lung V5**	**0.520, p < 0.001**
**Lung V10**	**0.504, p < 0.001**

**Lung V20**	**0.301, $p < 0.001$**
